# Role of transumbilical laparoendoscopic single-site surgery in the implementation ERAS in gynecology oncology: a retrospective study

**DOI:** 10.3389/fonc.2025.1483878

**Published:** 2025-04-22

**Authors:** Fanlin Li, Ying Zheng, Sijing Chen, Qiao Wang, Kana Wang, Fan Yang, Jianhong Liu, Na Wang

**Affiliations:** ^1^ Department of Gynecologic Oncology, West China Second Hospital, Sichuan University, and Key Laboratory of Birth Defects and Related Diseases of Women and Children (Sichuan University), Ministry of Education, Chengdu, Sichuan, China; ^2^ Department of Ambulatory Surgery Center, West China Second Hospital, Sichuan University, and Key Laboratory of Birth Defects and Related Diseases of Women and Children (Sichuan University), Ministry of Education, Chengdu, Sichuan, China

**Keywords:** ERAS, TU-LESS, gynecology oncology, cervical cancer, endometrial cancer, ovarian cancer

## Abstract

**Objective:**

The aim of the study was to verify the fast recovery effect of transumbilical laparoendoscopic single-site surgery by analyzing the operative and postoperative outcomes of patients with various gynecological malignancies in implementing The Enhanced Recovery After Surgery (ERAS) protocols.

**Design:**

A retrospective study.

**Setting:**

A university academic hospital.

**Population or sample:**

Patients with cervical, endometrial or ovarian cancer undergoing transumbilical laparoendoscopic single-site surgery by a single experienced surgeon.

**Methods:**

This was a retrospective consecutive single-center study of patients with cervical, endometrial, or ovarian cancer undergoing transumbilical laparoendoscopic single-site surgery for full surgical staging from November 2017 to January 2022.

**Main outcome measures:**

The main outcomes were the perioperative outcomes in various surgeries, including surgery time, estimated blood loss, length of hospital stay, and complications.

**Results:**

315 gynecologic malignant cases successfully experienced transumbilical laparoendoscopic single-site surgery (TU-LESS) between November 2017 and January 2022 in West China Second Hospital were incorporated, including 195 cervical cancers, 85 endometrial cancers and 35 ovarian cancers. The average age for patients is 47.48 (SD = 8.77). 152 (48.25%) patients have a history of previous pelvic and abdominal surgery. The average operating time and blood loss are 273.71 (SD = 87.12) minutes and 166.87 (SD = 237.09) ml, respectively. The average time for the first passage of flatus is 43.68 (SD = 29.75) hours. The hospitalization is 5.30 (SD = 2.42) days on average.

**Conclusions:**

TU-LESS can enhance the recovery of patients who suffer from gynecological malignancies by implementing ERAS with fast flatus, less pain, shorter hospitalization and better rehabilitation.

## 1 Introduction

The Enhanced Recovery After Surgery (ERAS) pathway was designed to improve the recovery of surgical patients through the preoperative, operative, and postoperative stages (1). This evidence-based protocol provides active strategies to minimize the stress of surgery, reduce postoperative complications, decrease hospital stay, and facilitate improved functional recovery (2, 3).

Morbidity associated with surgery affects patient outcomes, quality of life, and survival rates (4). Although most investigations of ERAS protocols are implemented in open surgery, patients who undergo laparoscopic surgery can also benefit from following the ERAS protocols, according to multiple studies (1). Numerous reports have demonstrated that operative and postoperative complications can interfere with overall survival rates (4). A minimally invasive surgical approach can also mitigate the immunological stress of surgical insult (5).

Transumbilical laparoendoscopic single-site surgery (TU-LESS) is an emerging technique that could provide another option for minimally invasive surgery in gynecologic surgery (6–8). TU-LESS has some advantages compared to conventional laparoscopy, including quicker recovery, reduced pain, a shorter hospital stay, minimal injury, and good cosmetic outcomes (9, 10). TU-LESS is an advanced technique in which the surgeon places surgical instruments through a single, small incision in the umbilicus to perform the procedure. Compared with conventional laparoscopy, once the proficiency and competency of the surgeon are achieved, TU-LESS is capable of minimizing postsurgical complications, for example, organ damage, postoperative infection, and pain to the most degree (11).

The purpose of this study was to investigate the role of TU-LESS in the implementation of the ERAS program in gynecologic oncology. We examined perioperative outcomes, including length of hospital stay, perioperative complication rates, and readmission rates in patients undergoing TU-LESS at the West China Second Hospital.

## 2 Materials and methods

### 2.1 Patient population

Institutional Review Board approval was obtained to retrieve data on patients undergoing transumbilical laparoscopy single-site surgery at our department. Of the 315 cases with gynaecological malignancies conducted between November 2017 and January 2022, the surgical outcomes were retrospectively reviewed. All data was collected from West China’s second hospital database. One single experienced surgeon conducted all operations.

#### 2.1.1 Inclusion criteria

- Diagnosed with cervical cancer, endometrial cancer, or ovarian cancer.
- Underwent TU-LESS for comprehensive surgical staging or diagnostic surgery between November 2017 and January 2022.
- Aged between 18–75 years with BMI ≤ 30 kg/m².
- No severe comorbidities contraindicating laparoscopic surgery.

#### 2.1.2 Exclusion criteria

- Patients with poor nutritional status or severe malnutrition.
- Patients with Acute Infections or Uncontrolled Diseases.
- Patients with Severe Psychiatric Disorders.
- Emergency Surgery.
- Concurrent malignancies or synchronous tumors.
- Previous pelvic radiotherapy.

### 2.2 Data collection and definitions

Data collection included patient age, BMI, medical comorbidities, and surgical history. The surgical procedures were classified and counted according to the type of the cancer, including extrafascial hysterectomy, modified radical hysterectomy, radical hysterectomy, and others. Modified radical hysterectomy is defined as the removal of more parametrium than extrafascial hysterectomy while preserving the blood supply to the distal ureter and bladder. The ureter is separated from the ureteral tunnel. The broad ligament is transected at the level of the ureter, with partial transection of the sacral ligament, and the bladder is partially mobilized. A vaginal resection of 1-2 cm is performed. Operative outcomes included surgery time, estimated blood loss, length of hospital stay, and conversion to multi-port laparoscopy or laparotomy. *Surgical time* was defined as time from skin incision to closure. *Intraoperative complications* were defined as vasculature, gastrointestinal and nervous injury. Postoperative complications included infection, embolism, fistula, intestinal obstruction or readmission. Postoperative fever was not identified as a sign of infection if it occurred within postoperative day five (12). We newly standardized an enhanced recovery index (ERI) to evaluate patients’ recovery. If patients have removed the catheter (cervical cancer excluded) with unblocked urination, temperature is normal, and flatus is done, they can discharge. We defined this ERI metric as the number of days required to meet the criteria as mentioned above.

### 2.3 The enhanced recovery after surgery protocol

**Figure 1:**
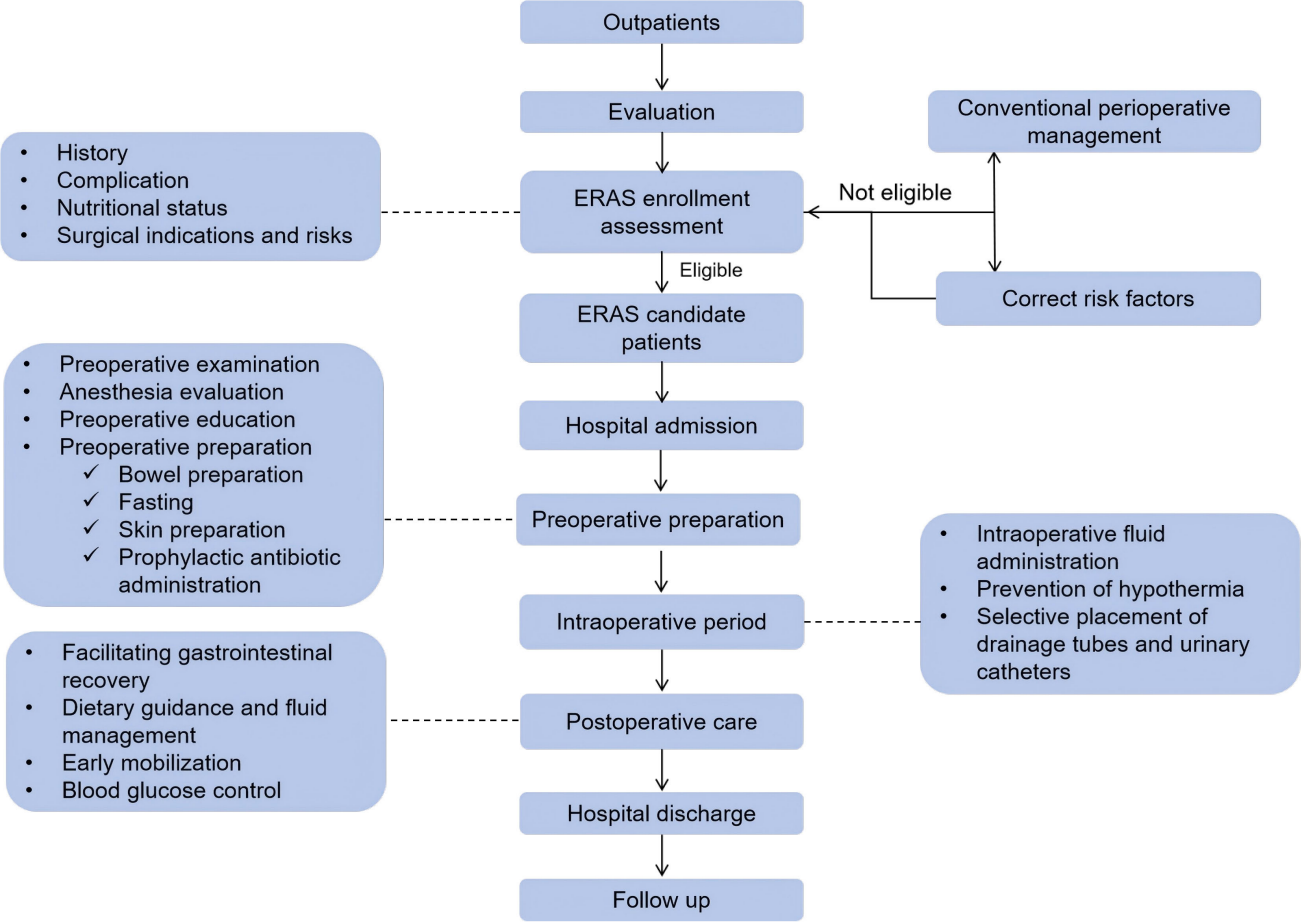
Enhanced Recovery After Surgery (ERAS) protocol.

According to the “Chinese Expert Consensus of Accelerated Rehabilitation of Gynecological Surgery” in 2019, to reduce the incidence of intraoperative and postoperative complications, promote postoperative rehabilitation, shorten the hospitalization time, and reduce the burden of patients. We formulated the “Accelerated Rehabilitation of Gynecological and Reproductive Endocrinology Surgery Patients Standard Operating Procedure”, as follows (**Figure 1**):

#### 2.3.1 Preoperative optimization measures after admission

##### 2.3.1.1 Preoperative education

After admission, the hospitalization procedures, safety, preoperative preparation, perioperative treatment procedures (including surgery and anesthesia), steps for the patient to complete, postoperative rehabilitation, pain management, discharge standards and other contents are introduced in detail.

##### 2.3.1.2 Risk screening and prevention of venous thromboembolism syndrome

Patients are screened for the risk of venous thromboembolism syndrome (venous thromboembolism, VTE), and taking corresponding preventive measures according to the risk level can effectively prevent the occurrence of VTE.

##### 2.3.1.3 Bowel preparation

Mechanical bowel preparation only applies to a. Surgeries above grade III; b. patients with deep pelvic endometriosis; c. patients with severe constipation. Methods: Choose oral laxatives or clean enemas and choose antibiotics.

##### 2.3.1.4 Shorten the preoperative drinking ban and fasting time

- No drinking time: delayed to three hours before anesthesia. Three to six hours before anesthesia, clear drinks can be taken orally, but cannot contain alcohol, and the total amount does not exceed 200 ml.
- Fasting time: fast starchy solid food and dairy products from six hours before anesthesia.

##### 2.3.1.5 Prophylactic use of antibiotics

Class II incision requires prophylactic antibiotics with intravenous infusion thirty minutes to one hour before incision.

#### 2.3.2 Optimization measures during the operation

##### 2.3.2.1 Surgical method

Try to complete the operation under the concept of precision, minimally invasive, and injury control to reduce traumatic stress and promote postoperative recovery.

##### 2.3.2.2 Optimize intraoperative fluid rehydration

The preferred balanced salt solution can reduce the occurrence of hyperchlorinated metabolic acidosis. 1~2L of balanced salt solution was given during the operation according to the patient’s blood pressure, breathing rate, and heart rate.

##### 2.3.2.3 Placement of abdominal drain

The drainage tube is not routinely placed. In the case of surgical wound infection, poor blood transport, or other adverse factors affecting the incision healing, the indwelling drainage tube can be considered, but it should be removed as soon as possible after surgery.

##### 2.3.2.4 Placement of the urinary catheter

The urinary catheter is retained according to the intraoperative conditions but removed immediately after surgery.

##### 2.3.2.5 Prevention and treatment of PONV

Postoperative nausea and vomiting (PONV) is a common issue following general anesthesia. Recommended preventive measures include anesthesia induction and maintenance of propofol, use of volatile anesthetics as appropriate or in reduction, minimization of postoperative opioids, and avoidance of fluid overload. 5-HT receptor antagonists are first-line drugs with compound low-dose dexamethasone (4 to 8 mg).

#### 2.3.3 Optimization measures after surgery

##### 2.3.3.1 Controlled fluid infusion

After awake anesthesia, patients can drink warm boiled water from 10~15 ml/h to 6 hours after exhaustion, and eat general food after defecation. For patients with gynecological malignant tumors, including patients undergoing intestinal resection and anastomosis, the diet transition can start within 24 hours after surgery.

##### 2.3.3.2 Multimodal analgesia

According to the situation, local anesthetic infiltration, transversus abdominis plane (TAP) block combined with low-dose opioid patient-controlled intravenous analgesia or NSAIDs analgesia regimen can be selected.

##### 2.3.3.3 Postoperative anticoagulation therapy

Patients with a high risk of VTE should continue anticoagulation therapy after surgery, and low molecular weight heparin combined with elastic socks or intermittent inflatable compression pump may be considered. Low Molecular Weight Heparin (LMWH) is recommended until 28 days after surgery in patients who received laparotomy.

#### 2.3.4 Discharge criteria

- Basic discharge criteria: recovery of semi-liquid diet; stop intravenous rehydration; oral analgesic drugs provide good analgesia; good wound healing, no signs of infection; good organ function status and free movement.
- Individualized discharge criteria: individualized discharge criteria should be formulated based on the patient’s condition and postoperative recovery.

#### 2.3.5 Follow-up visit

All patients with cancers were arranged to evaluate the pathologic results 2 weeks after surgery to assess if adjuvant therapy was essential to achieve therapeutic effect. The pathological results and staging status were recorded based on the FIGO (International Federation of Gynecology and Obstetrics) staging system: the 2018 edition for cervical cancer, the 2009 edition for endometrial cancer, and the 2014 edition for ovarian cancer. Follow-up visits were scheduled every 3 months in the first 2 years and at 6-month intervals in the subsequent 3 years.

### 2.4 Statistic analysis

Statistical analysis was performed using the SPSS version R26.0.0.0 and Graphpad Prism. Categorical factors were summarized using frequencies and percentages, while continuous measures summaries used means and standard deviations.

## 3 Result

### 3.1 Patients’ characteristics

Patient characteristics were shown in **Table 1**, and **Tables 2** and **3** stated perioperative outcomes. All the pathologic outcomes in 3 cancers were detailed in **Table 4**.

**Table 1:** Demographic characteristics of patients in three types of cancer.

Patient Demographics	Total (N = 315)
Demographics	
Age	47.48 (8.77)
Manifestations	
Irregularly vaginal bleeding	123
Contact bleeding	72
Excessive menstruation	7
Vagina discharge	11
Pelvic mass	8
Physical examination	67
Medical Comorbidity	
History of cancer	8
Hypertension	30
Diabetes	9
PCOS	3
Infertility	3
Surgical Comorbidity	
Laparotomy	114
Laparoscopy	51
Menopause	
Yes	124
No	191
Childbearing history	
Gestation	3.23(2.00)
Pregnancy	1.438(0.85)
Family history	70
BMI (kg/m^2^)	23.48 (3.64)

**Table 2:** Operative outcomes of patients in three types of cancer.

Operative outcomes	Total (N = 315)
Procedures	
Cervical Cancer	
Extrafascial hysterectomy + bilateral adnexectomy + PLND	37
Modified radical hysterectomy + bilateral adnexectomy + PLND	12
Radical hysterectomy + bilateral adnexectomy + PLND	133
Radical trachelectomy + PLND	1
Complete parametrectomy/upper vaginectomy + PLND	4
Diagnostics laparoscopy	8
Endometrial Cancer	
Extrafascial hysterectomy + bilateral adnexectomy + PLND	76
Modified radical hysterectomy + bilateral adnexectomy + PLND	6
Maximal tumor debulking surgery	3
Ovarian Cancer	
Comprehensive staging surgery	34
Diagnostics laparoscopy	1
Surgical time (minutes)	273.71 (87.12)
Blood loss (ml)	166.87 (237.09)
Surgical complications	
Vascular complication	9
Injury to the bladder	3
Injury to the ureter	2
Gastrointestinal Injury	3
Conversion	
Laparotomy	2
Laparoscopy	11
Hospital stay (days)	5.30(2.42)
Enhanced Recovery Index (days)	3.63(1.68)
First passage of flatus (hours)	43.68(29.75)
VAS pain score	
12	2.06 (0.94)
24	1.88 (0.87)
36	1.21 (0.77)

**Table 3:** Postoperative outcomes of patients in three types of cancer.

Postoperative outcomes	Total (N = 315)
Postoperative outcomes	
Pneumonia	1
Infection	9
Intestinal Obstruction	1
Paresthesia	3
Thrombosis	1
Lymphatic retention	1
Transfusion within 30 days	13
Delayed injury diagnosed within 30 days	
Urinary fistula	2
Incisional hernia	1
Vaginal cuff hemorrhage	3

**Table 4:** The oncologic outcomes of patients with cervical cancer^a^.

Oncology outcomes	Total (N = 195)
Stages	
I	
IA1	55
IA2	5
IB1	87
IB2	23
IB3	1
II	
IIA1	13
IIA2	1
IIB	4
III	
IIIC1	3
IV	
IVA	3
Pathology details	
LVSI	54
Adjuvant therapy	
Radiotherapy	49

a. The staging of cervical cancer is based on the FIGO 2018 edition.

We viewed 315 patients with cancers between November 2017 and January 2022, except 11 conversions to porous laparoscopy and 2 to laparotomy, while others successfully experienced total TU-LESS. The average age for patients is 47.48 (SD = 8.77). Moreover, the mean BMI is 23.48 (SD = 3.64). 152 (48.25%) patients have a history of previous pelvic and abdominal surgery. In cervical cancer (CC) patients, seventy-two patients complained of contact bleeding, eight patients complained of vaginal discharge, and fifty-seven patients were screened from physical examination. In endometrial cancer (EC) patients, forty-nine patients were admitted due to irregular vaginal bleeding, and five patients were admitted due to excessive menstruation. Fifty-three patients were complicated by hypertension, and 12 patients were equipped with diabetes mellitus. The mean BMI for EC patients was 24.26 (SD = 3.60). Forty-seven patients were postmenopausal. The mean gestation was 2.84 (SD = 1.96), and the mean pregnancy was 1.19 (SD = 0.90). With seven cases absent from tumor biomarkers in ovarian cancer, eighteen cases were reported with elevated tumor biomarkers.

### 3.2 Surgical outcomes

The TU-LESS surgery is exhibited in **Table 2**. A retrospective analysis of surgical interventions for gynecologic malignancies was performed. In cases of cervical cancer, thirty-seven patients underwent extrafascial hysterectomy with bilateral adnexectomy and pelvic lymph node dissection (PLND). Twelve patients with preoperative diagnoses of FIGO stage IA1 with positive lymphovascular space invasion (LVSI) and FIGO stage IA2 received modified radical hysterectomy with bilateral adnexectomy and PLND. Additionally, one hundred and thirty-three patients underwent radical hysterectomy with bilateral adnexectomy and PLND. Fertility-sparing surgery was performed in one patient with radical trachelectomy and PLND. Four patients who had previously undergone hysterectomy underwent complete parametrectomy with upper vaginectomy and PLND. Furthermore, eight diagnostic laparoscopies were conducted. For endometrial cancer, seventy-six patients underwent extrafascial hysterectomy with bilateral adnexectomy and PLND. Six patients with suspected cervical involvement received modified radical hysterectomy with bilateral adnexectomy and PLND. Maximal tumor debulking surgery was performed in three patients who were preoperatively diagnosed with type II endometrial cancer. In ovarian cancer, comprehensive staging surgery was carried out in thirty-four cases, while one patient underwent diagnostic laparoscopy.

Nine patients received diagnostic laparoscopic surgery to obtain para-aortic lymph nodes or retrieve a pathologic biopsy result for staging, which included eight cervical patients and one ovarian patient. However, the FIGO stage went through an upgrade to FIGO stage IIIC1p in two patients with cervical cancer. One patient was preoperatively diagnosed as IB1, and the other was primarily diagnosed with carcinoma *in situ*, but invasive cancer to be ruled out. Unfortunately, positive lymph nodes metastasis was found in pathologic results. The average operating time and blood loss are 273.71 (SD = 87.12) minutes and 166.87 (SD = 237.09) ml, respectively. The average time for the first passage of flatus is 43.68 (SD = 29.75) hours. The hospitalization is 5.30 (SD = 2.42) days on average. The average enhanced recovery index is 3.63 (SD = 1.68) days. The median number of pelvic and paraaortic lymph nodes removed is 25.42 (SD = 8.87). Thirty-one patients underwent para-aortic lymphadenectomy, and five of them accessed the infrarenal para-aortic region. Intraoperative complications occurred in thirteen cases, seven of which experienced vascular injury, and the rest of which presented three gastrointestinal injuries and three urinary injuries. All injuries were identified during the operation and repaired intraoperatively with conversions if necessary. There were eleven conversions to porous laparoscopy and two to laparotomy due to severe pelvic adhesion and intraoperative complications. VAS score presented as 2.06 (SD = 0.94), 1.88 (SD = 0.87), and 1.21 (SD = 0.77) at 12, 24, and 36 hours after surgery. Transfusions occurred in 13 (4.13%) patients. Postoperative complications appeared in 21 cases, including nine postoperative infections, three vaginal stump hemorrhages, one chronic pneumonia, one thrombosis, one incomplete intestinal obstruction, two urinary fistula, three paraesthesia and one lymphatic retention. A hernia was reported 2 months after surgery, and a herniorrhaphy was done.

One patient with cervical cancer who failed to remove the catheter after 3 months from discharge was reported. One patient with cervical cancer complained of a urinary fistula, which was repaired by additional surgical intervention. In endometrial cancers, 69 of 86 (80.23%) patients underwent surgical staging with pelvic lymphadenectomy, among which twenty-one patients experienced para-aortic lymphadenectomy. The rate of R0 resection reached 94.29% (33/35) in ovarian cancers. The overall rate of complications (transfusions and postoperative fever excluded) was 6.67% (21/315).

### 3.3 Pathologic outcomes

The postoperative FIGO staging results for all patients, categorized by tumor type, were listed in **Tables 4**–**6**. Upon examination of the postoperative pathology reports, it was found that squamous carcinoma was the most common histological type of cervical cancer (one hundred eighteen cases). There were thirty-three adenocarcinomas, five adenosquamous carcinomas, four neuroendocrine carcinomas, one carcinosarcoma, sixteen focal carcinoma formations, and two reported invasive carcinomas. However, detailed pathological descriptions were lacking in nineteen cases.

**Table 5:** The oncologic outcomes of patients with endometrial cancer^a^.

Oncology outcomes	Total (N = 85)
Stages	
I	
IA	62
IB	9
II	2
III	
IIIA	4
IIIC	7
IV	
IVB	1
Pathology details	
Peritoneal wash	
Positive	4
Negative	13
NE	67
LVSI	20
Adjuvant therapy	
Chemotherapy	16
Radiotherapy	11

a. The staging of endometrial cancer is based on the FIGO 2009 edition.

**Table 6:** The oncologic outcomes of patients with ovarian cancer^a^.

Oncology outcomes	Total (N = 35)
Stages	
I	
IA	12
IB	1
IC	9
II	
IIA	1
IIB	3
III	
IIIA	2
IIIB	1
IIIC	6
Pathology details	
Peritoneal wash	
Positive	7
Negative	20
NE	8
LVSI	3
Adjuvant therapy	
Chemotherapy	29

a. The staging of ovarian cancer is based on the FIGO 2014 edition.

Among the cervical cancer cases, the majority—one hundred forty-seven cases—showed no involvement of the cervical-corporeal junction, with twenty-seven patients exhibiting no signs of stromal invasion. Conversely, in forty-one patients, the depth of stromal invasion had reached at least the deep third or even full thickness. Notably, lymph node metastasis was detected in seventeen patients. Fifty-five cases were reported with lymphovascular infiltration.

In endometrial cancer, adenocarcinoma (sixty-two cases) and mucinous adenocarcinoma (nine cases) were the most frequently observed histological types. There were two carcinosarcomas, five clear cell carcinomas, four serous adenocarcinomas, one undifferentiated carcinomas, and two dedifferentiated carcinomas. Lymphovascular infiltration was noted in twenty-one cases. Thirteen patients showed no evidence of myometrial invasion, while eighteen exhibited deep half-myometrial invasion. The majority of cases (sixty-seven) were free from cervical-corporeal junction involvement. And lymph node metastasis occurred in six patients.

The findings for ovarian cancer presented a varied landscape, including eight adenocarcinomas, one squamous carcinoma, three mucinous adenocarcinomas, five clear cell carcinomas, nine serous adenocarcinomas, one sarcoma, and eight cases of germ cell tumors. Six patients had cancer cells detected in their ascitic fluid. Lymph node metastasis was observed in four patients.

## 4 Discussion

In patients with gynecological malignancies who received TU-LESS, surgical outcomes were ideal. The rate of perioperative outcomes did not significantly elevate in consideration of the technical difficulty. Early rehabilitation was achieved despite the broad resection margin, considering only 9 patients received diagnostics.

Our study had several strengths. We used a lap protector at the surgical site to prevent the spread of tumors and retrieve the sample as a whole to lower the rate of dissemination and plantation. Moreover, our surgeon has invented multiple ways to implement the suspension of organs called “Zheng’s 4C suspension” (13), successfully solving the limitation in TU-LESS where proper exposure of the surgical field is diminished, which may lead to an unsatisfactory extension of excisions and, consequently, poorer prognosis.

Our study had some limitations. The efficacy of applying discharge time to evaluate the speed of patient recovery is compromised. In our institution, the discharge time is arbitrary according to the subjective wills of patients, which leads to the introduction of newly defined ERI to evaluate patient outcomes. Additionally, all medical interventions were performed by one skilled surgeon who has mastered the TU-LESS technique. This may enlighten people that TU-LESS is a better surgical technique that facilitates faster rehabilitation once surgeons have mastered it, have achieved proficiency, and have strided across the learning curve. The different surgical approaches and instrument crowding which is difficult for even skilled surgeons to adapt to.

Furthermore, the retrospective nature of our study and the intrinsic biases of this design limit its credibility and generalizability. Case-control studies and randomized controlled trials should be conducted to evaluate patients’ postsurgical outcomes after TU-LESS compared to those after conventional laparoscopy. In addition, we are actively accumulating more cases to expand our sample size, which will help strengthen the evidence and provide a more comprehensive assessment of the efficacy and safety of TU-LESS in gynecologic oncology.

Our reported improvements in postoperative complication rates and readmission rates align with findings previously reported by other surgical specialties with the implementation of ERAS (14). Multiple meta-analyses found that enhanced recovery strategies reduced hospital stays (15, 16). However, there were few reports on the role of TU-LESS in the implementation of ERAS for the treatment of malignant diseases. Our study is the first integrated report about applying TU-LESS in gynecological malignancies with a large sample.

We found that using TU-LESS enhanced our patients’ recovery, the surgical outcomes were consistent with previous studies (17–19). Due to the smaller incision size, patients usually experienced less pain, faster recovery, fewer incisional complications, earlier passage of feces, earlier out-of-bed mobilization, and shorter hospital stays than those with conventional laparoscopy (20).

While exploring the TU-LESS technique, we noticed some potential complications due to the reduced number of ports. Previous studies have illustrated that single-site laparoscopy is associated with a higher risk of hernia in the umbilical site (21, 22). This potential risk prompted us to suture the peritoneum and anterior rectus sheath through direct visualization for larger incisions. Combined with the anchoring suture technique our surgeon invented (23), the scar can barely be detected, as deeper wrinkles allow for better cosmetic outcomes. After tracking the results of every patient who underwent a TU-LESS procedure in our institution, the rate of umbilical hernia is 0.1% (24). In our study, only one patient who had cervical cancer experienced an umbilical hernia, requiring herniorrhaphy. In contrast to previous studies, the incidence of hernia in our study was significantly lower (25, 26).

The rate of postoperative complications was slightly higher than in previous TU-LESS reports since most reports focused on benign lesions or others focused on malignant diseases with a small sample size (2.7%-8.5%) (17, 27, 28). Fagotti et al. reported that with endometrial cancer surgery, the median operation time was 129 minutes, ranging from 45 to 321 minutes, and that estimated blood loss was 70 mL, ranging from 10 to 500 mL—since the majority of patients did not undergo lymphadenectomy. In contrast to our strategy of full comprehensive staging surgery, including lymphadenectomy, we believe that the difference observed is acceptable (29).

In patients with advanced cervical cancer, lymphatic metastasis is a significant prognostic factor and a critical determinant for radiotherapy target delineation. Surgical or minimally invasive lymph node sampling can determine the involvement of pelvic and para-aortic lymph nodes, and precisely identify the location and extent of metastatic lymph nodes. By integrating pathological findings with imaging modalities, surgical staging provides histopathological confirmation of lymph node involvement, enabling the precise design of radiation fields, including the need for extended field irradiation and radiation dose considerations. It reduces the likelihood of undertreating or overtreating patients, which could optimize outcomes (30). Additionally, the single-port approach resulted in minimal trauma and rapid recovery, allowing for the timely initiation of radiotherapy.

The use of analgesics allowed patients to achieve early mobilization (31). Our study decreased the 24-hour VAS score compared with a previous report (1.8 vs 3.7) (32). The highest VAS score commonly appeared eight hours after surgery; once the VAS score exceeded 7 and patients complained of unbearable pain, extra analgesics were administered. Decreased opioid use allowed for earlier bowel function and decreased postoperative vomiting and nausea. Proper pain control made patients more likely to experience improved quality of life outcomes, avoiding readmission caused by impaired functional recovery (33).

Ramirez et al. reported that minimally invasive radical hysterectomy is associated with a high rate of recurrence (34). They raised concerns regarding the safety of utilizing laparoscopy to treat cervical cancer compared with laparotomy. Considering these concerns, we selected patients with tumors smaller than 2 cm to the best of our ability, used a lap protector to protect the incision, and followed the no-touch principle, such as a closed vaginal incision approach (35). We attempted to suspend the bilateral cornu cervi and uterine fundus without a uterine manipulator to achieve the same results, by which we avoided squeezing the cervix with minimal handling of the tumor (36–38). A recent study including 709 patients with cervical cancer, found that laparoscopy was equally effective for patients with no prognostic risk factors compared to that of the open approach (39). Based on the peri-operative outcomes of our study, the findings suggest that LESS may be a thought-provoking approach for the management of selected cases of cervical cancer.

Future studies should investigate robot-assisted LESS (R-LESS), as it may address technical challenges that arise from conventional inline visualization and instrument crowding with increased dexterity and more accuracy to excise lesions (40–42). More evidence needs to be collected to evaluate the feasibility and safety of this technique in gynecologic oncology.

We recommend TU-LESS for the treatment of gynecologic malignancies to facilitate enhanced recovery, as the results of our study are promising. However, the long-term outcomes should be further investigated. Due to the short timeframe of this study and the resulting limitations, prospective multi-center studies with long-term monitoring should be conducted to further confirm the feasibility and safety of TU-LESS.

## Data Availability

The original contributions presented in the study are included in the article/supplementary material. Further inquiries can be directed to the corresponding author.
